# Alzheimer's disease and natural cognitive aging may represent adaptive metabolism reduction programs

**DOI:** 10.1186/1744-9081-5-13

**Published:** 2009-02-28

**Authors:** Jared Edward Reser

**Affiliations:** 1 Department of Psychology, University of Southern California, 16380 Meadow Ridge Road, Encino CA 91436, USA

## Abstract

The present article examines several lines of converging evidence suggesting that the slow and insidious brain changes that accumulate over the lifespan, resulting in both natural cognitive aging and Alzheimer's disease (AD), represent a metabolism reduction program. A number of such adaptive programs are known to accompany aging and are thought to have decreased energy requirements for ancestral hunter-gatherers in their 30s, 40s and 50s. Foraging ability in modern hunter-gatherers declines rapidly, more than a decade before the average terminal age of 55 years. Given this, the human brain would have been a tremendous metabolic liability that must have been advantageously tempered by the early cellular and molecular changes of AD which begin to accumulate in all humans during early adulthood. Before the recent lengthening of life span, individuals in the ancestral environment died well before this metabolism reduction program resulted in clinical AD, thus there was never any selective pressure to keep adaptive changes from progressing to a maladaptive extent.

Aging foragers may not have needed the same cognitive capacities as their younger counterparts because of the benefits of accumulated learning and life experience. It is known that during both childhood and adulthood metabolic rate in the brain decreases linearly with age. This trend is thought to reflect the fact that children have more to learn. AD "pathology" may be a natural continuation of this trend. It is characterized by decreasing cerebral metabolism, selective elimination of synapses and reliance on accumulating knowledge (especially implicit and procedural) over raw brain power (working memory). Over decades of subsistence, the behaviors of aging foragers became routinized, their motor movements automated and their expertise ingrained to a point where they no longer necessitated the first-rate working memory they possessed when younger and learning actively. Alzheimer changes selectively and precisely mediate an adaptation to this major life-history transition.

AD symptomatology shares close similarities with deprivation syndromes in other animals including the starvation response. Both molecular and anatomical features of AD imitate brain changes that have been conceptualized as adaptive responses to low food availability in mammals and birds. Alzheimer's patients are known to express low overall metabolic rates and are genetically inclined to exhibit physiologically thrifty traits widely thought to allow mammals to subsist under conditions of nutritional scarcity. Additionally, AD is examined here in the contexts of anthropology, comparative neuroscience, evolutionary medicine, expertise, gerontology, neural Darwinism, neuroecology and the thrifty genotype.

## Background

Alzheimer's disease (AD) is a central nervous system disorder that gradually increases in severity with age resulting in memory loss, behavioral changes and a decline in thinking abilities. Connections between individual neurons are destroyed in the AD brain due to the formation of neurofibrillary tangles (i.e., tangled strands of neural fibers within the bodies of neurons) and senile plaques (i.e., masses of dying neural material containing toxic protein called beta-amyloid) which obstruct, damage and lead to the eventual death of many neural cells [1]. The most pronounced features of the AD state are thought to be decreased cerebral blood flow and decreased cerebral metabolism [2,3] due to the fact that dead neurons do not require oxygen or glucose [4]. The tangles and plaques responsible for this accumulate in all adults advancing in age, but do so at an accelerated rate in those that will develop AD [4]. All humans begin to develop the neurological markers of AD during their early 20s and continue to do so throughout life, most towards clinically irrelevant degrees [5]. But why would these markers present in everyone? How could natural selection have allowed them to become so invasive and ubiquitous if they did not hold some sort of evolutionary significance?

Humans are not the only species that develop plaques and tangles and are also not the only species whose behavior can be affected by them [6]. AD-like neuropathology is known to occur in the species of several mammalian orders, including many species of primates, but has the potential to become more severe in neuropathological presentation and in the degree of functional impairment in humans [7]. The wide phylogenetic spread suggests that it responds to some selective pressure. Furthermore, the fact that a wide variety of mammals exhibit AD-like neuropathology suggests that the first mutations responsible for it may have arisen tens of millions of years ago. It will not be easy to determine exactly when or how the genes responsible for AD evolved; however, the present article will focus on exploring why. Framing AD as part of a strategy to save calories during the later stages of life may explain many facets of the disease, such as (1) why it shares many similarities with known responses to starvation, (2) why it resembles a neuroecological change, (3) why it selectively affects specific neuroanatomical areas and not others, (4) why it presents comorbidly with the metabolic syndrome, (5) why it has been tied genographically with the thrifty genotype, and (6) why such a psychiatrically conspicuous syndrome was not eradicated by natural selection.

## Presentation of the hypothesis

It is thought that analyzing disease states from an evolutionary perspective can ultimately do much to inform and influence medical intervention strategies [8]. The present article will employ this approach with Alzheimer's disease (AD) emphasizing that, because susceptibility genes for AD are so prevalent in human populations, the traits that characterize preclinical (or presymptomatic) AD must have been naturally selected. Because the average life span of contemporary human hunter-gatherers is around 55 [9,10], it is thought that prehistoric man would have lived nearly as long. Given that clinical AD is only rarely diagnosed before age 55 [11], it is clear that individuals would rarely have lived long enough to develop AD in our evolutionary past. Natural selection then, could only have acted on the preclinical or prodromal AD phenotype and, as in other disorders of aging [7], would not have had the opportunity to curtail the detrimental effects, which of course appear late in life.

Some theorists assume tacitly that AD presents too late in reproductive life to have been exposed to negative selection [12]. They realize that diseases that arise after individuals become infertile cannot limit the total number of offspring produced, and that evolution has little way of excising such diseases. However, many facts about preclinical AD indicate that the genes responsible for it must have been exposed to blatant selective pressure, well before their bearers reached reproductive senescence. Particularly, neuropathological changes begin in the early 20s and usually constitute a "heavy load" 10–20 years before the first behavioral symptoms of marked cognitive decline surface [13]. Several forms of neurophysiological and intellectual decline have been shown to begin in the first few decades in individuals that will develop AD [14]. In fact, well controlled studies have shown that individuals that carry susceptibility genes for AD exhibit lower levels of intellectual functioning throughout life and are more likely to drop out of high school by age 15 when compared to their matched peers [15]. In other words, because the genes that cause AD create conspicuous neurological and behavioral characteristics that present during reproductive age, they could not have been invisible to evolutionary forces.

Additionally, some evolutionary theorists find it hard to imagine that AD could have been adaptive because of low ability for autonomy and self-sufficiency seen in AD patients. Despite some impairment, individuals with preclinical AD are regularly capable of both domestic autonomy and professional achievement [5]. Importantly, AD usually only creates conspicuously maladaptive behavior in people that live far past the average terminal age, which is around 55, in foraging societies [16]. Moreover, medical advances in the last few centuries have artificially extended life by several decades, and most experts believe that this has given AD neuropathology the time necessary to develop to an injurious extent that would have been impossible in prehistoric times [17]. In other words, AD in its advanced, debilitating form may simply be the unnatural progression of natural brain aging changes, which would have rarely occurred in shorter-lived, ancestral populations.

It is important to emphasize that, on a cellular level, all of the changes seen in Alzheimer's disease are also found in normal aging adults [18]. The neurofibrillary tangles [19], senile plaques [16] and neurotransmitter changes [20] are each present in aged-matched normals, with the same regional preferences, but simply to a lesser extent [11]. It has been estimated that even those elderly individuals that show the least cognitive impairment in old age would eventually be diagnosed with AD if they were able to live to around 130 [21]. The genes responsible for this "inevitable transformation" exist in every one. This frames AD-like neuropathology as a continuous, polymorphic cognitive strategy, where clinical AD represents the extreme side of a continuum. Other researchers have also concluded that AD is not a qualitatively distinct, abnormal entity, but is instead at one extreme end of a spectrum of capability in old age within which a great deal of natural variation exists [22]. Like many other polygenic, continuous traits the mutations responsible for pronounced AD were likely maintained by balancing selection (specifically, environmental heterogeneity). In other words, they were kept in our gene pool because, as environmental resource conditions fluctuated, different genetic polymorphisms, or "multiple alternate alleles" were favored.

Today, the costs of AD are well-documented [23], but the defensive manifestations may be hidden because of discrepancies between our modern and ancestral environments. Many traits that are clearly maladaptive in the present are now thought to have been adaptive in the ancestral environment [24] and this situation is known as an "environmental mismatch." The science of evolutionary medicine attempts to identify, analyze and explicate these traits [25]. Researchers have identified many "pathological" conditions such as atherosclerosis, cardiovascular disease, cystic fibrosis, diabetes mellitus, and obesity, and have helped to show that despite the stigma today, genetic susceptibility to these diseases would have conferred adaptive benefits in prehistoric times [8]. Williams and Nesse [26] suggest that certain criteria must be met to conclude that a "disease" may have been adaptive in the past: it should be relatively prevalent, heritable, and susceptibility should vary within the population. AD certainly meets the first three criteria [18], but now it is important to show that the fourth criterion can be satisfied – that the benefits associated with the condition must have outweighed the costs.

A handful of articles have attempted to explain the existence of AD in terms of antagonistic pleiotropy [12,17], however, AD and the underlying neuropathology have not been analyzed in terms of evolutionary medicine and have not been firmly reconciled with evolutionary theory. Perhaps it can be argued that AD is utterly pathological, has no advantageous qualities and no natural history. This article will espouse an opposite view and explore the assertion that AD has been naturally selected and represents a preservative ecological strategy. Many articles in the last few decades have analyzed various forms of psychopathology (e.g., anxiety, bipolar disorder, depression, obsessive compulsive disorder) in terms of evolutionary theory and evolutionary medicine [27,28]. This burgeoning area of research is often referred to as "evolutionary psychopathology." The present author has written articles analyzing various forms of neuropathology using this approach and has called this area "evolutionary neuropathology [29-31]". Using this framework, the present article is prepared to explore many congregating sources of evidence that strongly suggest that AD is highly explicable under evolutionary theory.

### Neural Darwinism, skill and specialization

Clearly, prehistoric foragers with preclinical AD would not have the same cognitive capacity to learn from their environment that they had when they were younger. It is probable; however, that they would no longer need this capacity. For reasons to be explored in this section, the cognitive symptoms of preclinical AD may have complemented the aging process on the ancestral, African savannah.

A conspicuous trend has been documented for decades in the study of neuron number: many animals overproduce neurons in their youth and then, later in life, lose the unused, inefficient ones [32]. This kind of neuron loss is known to be controlled by programmed cell death, or apoptosis, which is also a major contributing factor to the neuropathology in AD [33]. One function of neuron death in aging animals is to remove neurons that have not made useful or sufficiently numerous connections [34-37]. For example, in mammalian populations, neural degeneration in segments that control necessary functions such as limb movement is very rare [38]. It is more common, however, in less behaviorally critical neural tracts. A young mammal's cortex is full of neural tracts that may never be critical, but it is not clear which will be needed and which will not until the animal has acquired life experience [39]. Several researchers have associated this process of "economization by elimination" with strong positive increases in reproductive fitness [39-42]. A good deal of ethological evidence supports the idea that as animals of many different phyla mature and age, intensive learning may become less important with time [43]. In fact, one major neurodevelopmental trend seen throughout the human lifespan is a compelling example of this. The metabolic rate found in the brains of children is far higher than in those of young adults, which are, in turn, far higher than in those of middle-aged adults [44].

In the first decade of human life, the cerebral cortex undergoes dramatic fluctuations in energy consumption. The metabolic rate of the brain increases rapidly from birth and begins to reach adult levels by age two. From four years of age until nine, the amount of energy spent in a child's brain far exceeds that of an adult [45]. During this period, the brain's neural architecture is characterized by "hyperconnectivity," where neurons of the cortex have formed many more connections (synapses) than will be kept. Many of these will be selectively eliminated [46]. At ten years of age, the brain's metabolic rate begins to decline until the late teens, at which point the levels of glucose utilization have reached adult values. This pattern of metabolic decline persists naturally, in everyone, until death [45]. Throughout the lifespan, neurons and synapses that are not used are "pruned," limiting behavioral plasticity and constraining what can be learned in the future [47]. Only the connections that are utilized are maintained, and this inevitable process is responsible for the dramatic windows for learning (e.g., second languages and musical instruments) that close soon after adolescence [46].

The sharp diminishment in cerebral metabolism in young adulthood is currently conceptualized in the literature as an evolutionarily mediated response to changes in life-history dynamics [46], but modern AD researchers appear surprised that further reductions occur with advancing age. These reductions, even in late life, should be seen as part of a natural process of continuing development. Young children are small and their metabolic demands are met by their parents, yet they need to learn rapidly in order to become ecologically competent [48]. They can afford a high cerebral metabolism because they benefit so greatly from the incessant thinking and learning that accompanies it. Once the individual becomes an adult though, they need not expend quite so much energy actively learning and analyzing. The adaptive value of extracting large amounts of information from their environment and carrying it with them through time has decreased. This is because, in adulthood, individuals should have already internalized much of the cultural and ecological information that they will need.

As aging progresses, it is still necessary to be able to learn new mental maps, new language skills and new techniques. However, because so much of what is learnable is already known, the usefulness of this capacity must continue to diminish with increasing age. If an animal is able to survive to reach full adulthood, then the functional conceptualizations that it made during development have been working and the animal must have internalized information in a way that is conducive to survival. In this case it should not be necessary to radically change a mental set that has proven to be effective. The profound neuron loss with age to the nucleus basalis [49], the gateway module for new learning in the brain, may help to ensure that a mature organism cannot reprogram its tried and true behaviors with new, untested learning. It is accepted that elaborate, new memories have a tendency to interfere with old ones in both laboratory and ecological contexts [50].

"Academic learning is often explicit: professors point out the things to be learned, and students try their best to memorize them. But most ordinary human learning is probably implicit. A hunter may teach young people how to track an animal, or how to kill and skin it. Most of the time such practical activities can be taught more easily by modeling than by explicit labeling. Many of the subtleties of hunting and gathering may not even have names"

-Moscovitch et al., 2007 [51]

As the quote above explains, in ancient times, a hunter-gatherer's cognitive resources were probably used (much more than they are in modern times) to analyze, refine and coordinate muscle activity. It is an interesting observation that most AD patients can walk, gesticulate, inflect, intonate, gesture, and move perfectly fine. This is due to the fact that their procedural memory, the ability to learn and implement behavioral skills at an automatic, unconscious level, remains very much intact [52]. Interestingly, the structures responsible for procedural memory, the striatum, putamen and caudate nucleus largely escape neuropathological load in old age [53]. As such, patients with AD can exhibit severe episodic, semantic and working memory deficits, but because of preserved procedural memory, are usually able-bodied and retain many of the habits, skills and implicit memories that they have honed during their lifetime [5]. In fact, most individuals with AD who were athletes can continue to perform admirably, usually only impaired by physical aging (e.g. muscle tone and cardiovascular endurance) but not mental aging [54]. If reflexes, motor praxes and coordination of movement are generally proficient in individuals with pronounced AD in their 90s, then surely they would have been adequate in our ancient, preclinical ancestors in their 30s, 40s and 50s.

The changes during cognitive aging tend to preserve crystallized intelligence (i.e., the ability to use known information to instruct behavior), over fluid intelligence (i.e., the ability to use new information to instruct behavior) [55]. Moreover, it has been shown that even though general IQ score remains somewhat stable, crystallized intelligence grows rapidly and fluid intelligence declines rapidly between 15 and 60 years of age [56]. The increases in crystallized intelligence are thought to correspond with increased number of life experiences, and the decreases in fluid intelligence are thought to correspond with decreasing metabolic output of the brain with age. For these reasons an aging individual may be less likely to benefit from constant learning and concerted analysis and more likely to benefit from being vigilant for those stimuli that elicit known behavior patterns. It seems clear that the accretion of crystallized intelligence (knowledge) makes it so that fluid intelligence (deliberation) is less necessary. After all, it is far less costly to inform behavior using neural connections that have already been made than to go through the trouble of creating new connections. As Matt Ridley [57] states,

"Experience causes the unnecessary connections to wither away and thereby turns the brain from a general to a specific device. Like a sculptor chipping away at a block of marble to find the human form within, so the environment strips away the surplus neurons to sharpen the skills of the brain."

Brain development happens throughout life and is characterized from even a very early stage by two complementary processes, selection and instruction [58]. Both processes underlie learning and memory and are fundamental to cognition as we know it [59]. In selection, the synapses and neurons that are utilized most frequently are "selected" to be preserved, and those that are not used degenerate [60]. This process of neural selection, also called "neural Darwinism," is thought to be nearly as important as "instruction," which stands for the family of cellular and molecular processes that accomplish long-term potentiation [57]. The main difference between selection and instruction is that the latter necessitates biological resources. Preclinical AD can be viewed as a strategy that relies heavily on selection rather than on instruction – emphasizing a cheap and metabolically thrifty way to program behavior. The importance of metabolic thrift during human aging is what we turn to in the next section.

### Advancing age and the threat of starvation

Data from physical anthropology have shown that, in all foraging groups studied, elderly hunter-gatherers implement low-yield foraging strategies and procure food (measured in calories) at a rate that is far below that of younger foragers [61]. These aging individuals have more difficulty procuring foodstuffs than their younger counterparts do because, due to natural senescence, they can no longer meet the associated physical and athletic demands [62]. Unlike any other species, however, human foragers engage in food sharing, which allows the elderly individuals to live longer than they would be able to if they were forced to meet 100 percent of their own metabolic demands [62]. Traditionally, the older individuals in foraging groups seek out food on their own, but also rely heavily on younger individuals to supplement their diet [61]. This is an imprudent strategy because, during times of hardship when food was scarce, they would most certainly be threatened by starvation. We know that the Pleistocene era (roughly 2 million to 12 thousand years ago) was marked by frequent, prolonged, dry spells [63] and consequent widespread nutritional scarcity that continually threatened our ancestors [64]. It is clear that the demographic most susceptible to starvation during tough times, the aged, would have benefited the most from metabolic penny-pinching.

Food production, in total calories attained, in male hunter-gatherers increases dramatically every year between the ages of 16 and 25; this reflects the long learning curve for hunting ability [65]. The ratio of production to consumption (i.e., catching versus eating) hits the maximum at 25. At this point the individual is able to procure more food than it eats, and it shares the leftovers. This state of self-sufficiency plateaus and remains stable as late as age 45, after which time it drops precipitously for many years until death [61]. Food production ability eventually drops below food consumption resulting in insufficiency. This race for calories against the clock- a very foreign concept today even in developing nations- explains why aging humans desperately needed to conserve energy resources. In contrast, chimpanzee subsistence during aging is very different. Indeed, chimpanzees live their entire lives without food production ever dropping below consumption [61]. Because young chimpanzees don't share food with the older ones, old chimps, like most other wild animals in general, starve to death relatively soon after their food production ability begins to decline [66]. Human hunter-gatherers on the other hand exhibit a pronounced drop in food consumption – allowed of course by a diminishment of metabolic rate – that helps them to survive the drop in production [62].

Because elderly foragers cannot gather enough calories to meet the energy requirements of a younger forager, we can infer that they would have benefited from particular metabolic alterations that helped them cut down on energy expenditure. Fittingly, decades worth of gerontological research shows that elderly humans exhibit lower resting metabolic rates and that they accomplish this by down-regulating a number of physiological systems resulting in lower muscle mass, lower thyroid levels, lower growth hormone levels, lower testosterone levels and insulin resistance [67,68]. These physiological changes that occur with age suggest that natural selection is responsible for selecting our species for "advanced-age thrift," allowing elderly individuals to survive on smaller amounts of food [67]. Again, each of these thrifty metabolic changes that occur with age can be interpreted as pathological in the present yet adaptive in the past, and clearly represent a type of "senile plasticity" that permitted metabolic frugality [69]. This plasticity towards energy conservation presents so dependably in various tissues and organs that we might expect it to generalize to the brain as well.

## Testing the hypothesis

### Neuroecology and Alzheimer's

A growing body of literature has shown that neural tissue is highly metabolically expensive and that species with large brains have been forced to negotiate tradeoffs leading to compensatory adaptations in various organ systems [70]. Considering that humans spend 20–25 percent of their total resting energy budget in their brains alone – most primates spend between 8–9 percent [71] – it is clear that, as highly encephalized primates, humans must have been forced to make many inventive compromises [72]. This line of reasoning makes it seem sensible that any excusable opportunity to attenuate the number of calories consumed by the brain would increase an individual's chances of surviving periods of prolonged scarcity.

The emerging discipline of neuroecology has amassed a great deal of data (on an assortment of species, avian and mammalian) that demonstrate that, because the brain is very costly to maintain, specific brain regions can respond plastically to the environment, lowering energy consumption in response to deprivation and adversity [73]. It has been shown that many species have this adaptive ability to attenuate energy metabolism in the neocortex and hippocampus [74]. The animals have been naturally selected to accomplish this despite the fact that, due to the associated cognitive disadvantage, they are forced to employ less cognitively rigorous and lower yielding foraging strategies [75,76]. In fact, studies have shown that the hippocampus can adaptively vary both metabolic rate and volume with ecological rigor [73,76]. Birds and mammals have been shown to decrease energy expenditure in the hippocampus in response to environmental deprivation [77], season of low food availability, and decreased need to forage [76]. These findings have been interpreted widely and emphatically as examples of protective changes that can significantly increase the animal's ability to survive periods of nutritional scarcity [73]. Fascinatingly, the hippocampus is known to show, by far, the most severe decrement in metabolism when compared with other brain regions in AD [78-80].

Hippocampal atrophy and glucose hypometabolism in the neocortex are the two most well documented findings in AD [81], even in the early stages [82]. In fact, the postmortem diagnosis of AD concentrates primarily on the extent of neurodegeneration in the fronal cortex and hippocampus [83]. The fact that the hippocampus and the neocortex are the most affected areas in both the naturally aging brain and the AD brain [84] further suggests that the "neuropathology" represents an adaptive neuroecological change. This seems especially likely given that old age is strongly associated with a diminished foraging capacity in human hunter-gatherers [62]. The dentate gyrus is a subregion of the hippocampus that is highly ecologically significant in that it is the main area in the brain that generates new neurons throughout life. It is also the area of the hippocampus that is most damaged in normal aging and AD [5]. Perhaps there are unexplored ecological implications here! Furthermore, the hippocampus and the neocortex are known to have exhibited highly accelerated growth during human evolution [49] and thus the age-associated atrophy they exhibit in AD may signify that aging individuals can benefit from shifting towards more rudimentary, less cognitively rigorous, food extraction methods reminiscent of those used by our evolutionary predecessors.

The neuroecological literature informs us that hippocampal hypometabolism in animals invariably results in an alternate, less rigorous, ecological strategy and, like AD, is often accompanied by other physiological modes of energy conservation [74]. Overall cerebral metabolism in AD patients is much lower than in age-matched normal subjects [3], and the degree of severity of AD has been shown to be positively correlated with decreases in cerebral metabolism [2]. All of these facts are consistent with our characterization of AD "neuropathology" as a subtle but progressive neuroecological program that has been engineered by natural selection.

### The selectivity of neuropathological changes in Alzheimer's

It is known that neurodegeneration in AD is not at all uniformly distributed in the brain. Specific neuroanatomical structures are targeted by the disease relative to others [85], and if AD pathology represents a form of plasticity that was adaptive in our evolutionary past, we should expect to find evolutionary significance in the distinctively patterned distribution. The findings reported here are consistent with the idea that AD selectively diminishes the metabolic costs of neural areas that might prove extraneous to a deprived forager while sparing the essential areas.

AD pathology is most damaging to areas responsible for higher-order learning and explicit memory but spares areas essential for sensing and moving. For example, plaques and tangles are known to be much more prevalent within the anterior (learning) but not the posterior (sensory) cortex [85]. Scanning studies demonstrate reduced glucose utilization in the frontal, parietal and temporal association cortices of patients with Alzheimer's disease, when compared with age matched controls, whereas the primary sensory, motor and visual regions are preserved [49]. In fact, the sensorimotor and visual cortex have been shown to suffer the least damage of any cortical structures [86] and their selective preservation has been described as "perplexing." The sensorimotor and visual regions are areas that, if damaged, would certainly disrupt the abilities to see and move. In other words, within the cortex, Alzheimer's follows a genetic program which selectively spares the areas associated with the senses and muscle movement – regions that any foraging animal would find indispensable. It primarily affects the higher areas that are associated with lofty but expendable, abstract thought.

Within each of the major cortical structures, the higher-order "association cortices" are the most vulnerable to plaque and tangle formation whereas the adjacent primary sensory areas are the least vulnerable [87]. The association areas are responsible for functions that one can imagine might be superfluous to a weathered forager that has already "seen it all" such as mental comparison, conceptual integration and analytical thought [58]. The primary sensory areas though, which are highly conserved, allow basic perception and stereotyped response to stimuli [87]- functions indispensable for any animal. Genes that promote damage to such vital areas would be so damaging to reproductive fitness that we can see why they would not be maintained and why they would not exist today. Genes that promote damage to less vital, "metacognitive" areas would be more likely to persist, especially given that these areas are responsible for abstract learning [58]- which is, of course, very important to a youngster, but less important to the experienced aged. It is also interesting to mention that, phylogenetically, association cortices expand at a faster rate than primary sensory cortices [88], indicating that, in cortical function, the AD brain operates like an evolved throwback rather than in some arbitrary fashion.

The cortex, which is proportionally very large in humans and tiny in the simplest vertebrates, is the area of the brain that is damaged the most by AD [86]. The noncortical areas responsible for respiratory and cardiac function, waking state, emotionality, sensory perception and motor capacity are relatively spared by AD; the damage that does occur only begins to compromise these functions in very late, advanced AD. All noncortical areas display proportionately far less damage than the frontal cortex and hippocampus [81]. These two, phylogenetically new, areas are thought to have allowed humans the patience and analytical ability to learn cognitively demanding food extraction techniques and social conventions [89,90]. Interestingly, the noncortical brain regions that do exhibit neuropathology are primarily regions that have undergone very recent "integrated phylogeny" (i.e., extensive elaboration with association neocortices since our divergence with the other apes) [49]. These areas include the posterior hippocampus, the entorhinal cortex, the basocortical amygdaloid complex and the nucleus basalis of Meynert. The fact that these findings reveal reverse-integrated phylogeny has influenced authors to posit that Alzheimer's is, neurologically, a phylogenetic disease [49]. This characterization fits neatly with the present hypotheses and further frames AD not as arbitrarily localized pathology, but as an atavistic cerebrotype that recapitulates a fundamental, ancestral configuration.

The relative deficiencies of the preclinical AD brain may be seen as mimicking the mental faculties of a lower primate. The facets of cognition that are struck the hardest by AD include: sustained attention, prolonged concentration, inhibition of impulse and higher order capacities for deliberation, prudence and forethought. Apes are generally seen as being less deliberate, prudent and inhibited, and as having less capable working memory [91]. These "relative deficits" do not detract from the fact that apes are exquisitely crafted, able-bodied animals whose minds are maximized for their ecological niche. The main way that the ape brain is different from our own is in size of the cortex, and thus their limbic (emotional) system is proportionately large [88]. Consistent with all of this, it has been found that the cortical areas that are the least vulnerable to AD are the ones with the smallest connectional "distance" (measured in synapses removed) from the limbic areas [87]. Interestingly, this same pattern is also true of normal brain aging, indicating that all of us are designed to make very precise, very deliberate neurological changes with age. Relative selectivity in the AD brain should be taken as a record of our behavioral past, where distribution of neuropathology evinces relative adaptive value of individual processing modules and, thus, also of individual cognitive processes.

Several experiments using a variety of complex tasks have shown that the level of metabolic activity in the cortex drops with practice, familiarity and increasing automaticity [58]. Many different tasks that require concentration, executive activity and recruitment of various brain areas when the task is new can be automated with experience to a point where the task can be completed efficiently with only a minimal expenditure of metabolic activity [92]. Many tasks, even ones that seem relatively complex and unpredictable, such as playing the videogame Tetris, show this effect [93]. One might imagine that the tasks associated with foraging could easily become automated in the same way and that the drop in cortical metabolic activity with age represents a cunning move on the part of natural selection. Because the metabolic requirements for the completion of familiar tasks decreases with age and experience, aging individuals can afford to lower overall cerebral metabolic output and still maintain their fundamental behaviors and their ability to interact adeptly within their environment.

### Comparative physiology and Alzheimer's

Definitive proof of the adaptive value of a physiological phenomenon can be very hard to attain, but the comparative approach is thought to provide strong evidence [94]. This approach commonly compares the same physiological process in (1) closely related species that live in strikingly different environments, or (2) distantly related species that live in identical environments. If the process is associated with adaptation to environment, it is expected that group 1 will show large variation in the trait and group 2 will show little. First we will employ approach number one and examine the large natural variation in susceptibility to AD within our species.

Two human groups that differ tremendously in way of life and natural environment from other humans are the Khoi San hunter-gatherers and the pygmy foragers of sub-Saharan Africa. The APOE 4 allele (currently the gene most strongly associated with AD susceptibility) has been shown to be more than twice as common in Khoi San (.37) [95] and pygmies (.40) [96] as it is in other populations, where it is usually less than 20 percent and often less than 12 percent. Why might this be so? These individuals represent human groups that, because they never utilized agriculture, have been exposed to nutritional shortages up until recent times [97]. They live and forage in much the same way that their ancestors must have for tens of thousands of years. The fact that the APOE 4 gene is so frequent among these groups suggests that there is a strong relationship between way of life, nutritional scarcity and selective pressure on AD.

Modern molecular studies have shown that the Khoi San are more distantly related to the rest of humanity than any other group, meaning that they may have changed the least since we all diverged from a single, ancestral "stem" group around 180,000 years ago [98]. This suggests that the ancient foraging ancestors to us all may have manifested preclinical AD to a larger degree than the modern human population at large. Today, the incidence of APOE 4 remains highest among all populations where an economy of foraging still exists (e.g., Pygmies, Khoi San, aborigines of Malaysia and Australia, Papuans, European Lapps, and some Native Americans), or the food supply is known to have been frequently scarce, or nutritionally poor [97]. This data shows that AD does in fact show high variation between closely related but ecologically disparate groups. In the next section of this article we will consider the observation that known "thrifty" genes, associated with adaptive energy conservation, show this same geographical pattern, strengthening the association between AD and metabolic thriftiness.

Now we will consider the second comparative approach; a commonality in two distantly related species, rats and humans. Animals that have been subjected to starvation or nutritional deprivation exhibit multiple physiological methods of energy conservation [99]. These changes involve lowering the energy demands of the most expensive organs. Interestingly, some of these changes are known to occur in the brain, and some appear highly analogous to the changes seen in AD. One of the neurohistological hallmarks of AD, hyperphosphorylated tau proteins, form reversibly in the brains of starving or stressed rats. After a number of hours of food restriction, hyperphosphorylated tau begins to accumulate within neurons of the rat hippocampus [100]. This abnormal tau, a transport protein, is one of the primary pathohistologicall hallmarks of AD, and is the cause of neurofibrillary tangles in humans [101]. Kurt Heininger [102] has concluded that these molecular similarities shared between AD and starving mammals suggests that AD constitutes a "rescue program" that "actively adapts to progressive fuel deprivation."

In rats, the accumulation of these proteins causes pronounced decrements in brain metabolism allowing the rat more time to seek out food before it collapses from starvation. This phenomenon, which has been replicated reliably, has been explicitly attributed adaptive significance [103]. Interestingly, after the rat is fed again, the tau dephosphorylates and the hippocampus regains normal function [100]. This reversible, phenotypic change in rats is analogous to the permanent change seen in AD because both may protect against starvation. Before these changes were observed in starving lab animals, they were thought to be specific and exclusive to AD. Ironically, the literature today views these changes as adaptive alterations to starvation in lab rodents [103,104] but as pathological in AD.

Mammals are well-known to demonstrate a suite of plastic responses to extreme hunger and starvation that help to minimize energy expenditure. The most dramatic of these includes suppression of metabolic rate, reduction of thyroid hormone levels and growth hormone levels, suppression of gonadal function, and an increased activation of the hypothalamic-pituitary-adrenal axis [99,105]. As discussed in the next section, aging individuals and especially individuals with AD exhibit increased incidence of each of these physiological alterations [68,106]. Ostensibly aging humans in the environment of evolutionary adaptedness would have benefited from the same physiological alterations as starving animals. This further characterizes AD neuropathology as a response to nutritional scarcity.

Many species of mammals, most notably primates, have been shown to develop the neuropathological hallmarks (both senile plaques and neurofibrillary tangles) of AD [7,107] It has been hard for specialists to link changes in these animals' behavior to the underlying pathology because they are wild. However, our domesticated cats and dogs, whose lives have also been artificially extended in the last few centuries, also exhibit Alzheimer's like neuropathology. Cats, dogs and some other mammalian pets diagnosed with cognitive dysfunction syndrome (CDS), which is thought to be highly comparable behaviorally to Alzheimer's in humans [108], have been shown to have specific molecular and cellular changes that mimic the pathophysiology of AD [109]. In fact, the behavioral abnormalities of CDS in cats and dogs are accompanied by the same hallmarks of AD in humans: high levels of diffuse amyloid beta accumulation as well as hyperphosphorylated tau, predominantly in cortical and hippocampal areas [110]. These findings suggest that AD-like neuropathology may have played an adaptive role in the life histories of our pet's non-domesticated ancestors. Perhaps most long-lived mammals can benefit from diminishing the expenditure of resources on cognition during the aging process. Very short-lived mammals, reptiles, amphibians and fish generally do not show clear signs of AD pathology [7], and this may be because their brains are far smaller, their learning arcs are much abbreviated, their life spans are typically much shorter and their life histories are much different. Perhaps the degree of expression and the severity of the symptoms increased in humans (relative to other mammals) because of life history parameters characteristic of our species, such as large intergenerational resource flows, large brains and long lifespan.

### Alzheimer's and the thrifty genotype

The human brain is an extravagantly expensive organ [70]. In fact, the mass specific metabolic rate of brain tissue is over 22 times that of skeletal muscle [111]. Such a ravenous organ can be viewed as an unfavorable encumbrance because it is known that the Plio-Pleistocene was marked by recurring ice ages where large, polar glaciations would frequently cause the African savannah to become arid, hot and nutritionally scarce [64,112]. Such unpredictability of food resources in our environment of evolutionary adaptedness is widely thought to have seriously impacted the human genome [113]. In fact, the blossoming science of "evolutionary medicine" has pointed out that many human diseases are simply vestiges of our hunter-gatherer existence and some of the best characterized among these are thought to have provided protection against starvation [8]. Genetic tendencies toward adiposity and insulin resistance would have helped our metabolisms deal with low food availability in the ancestral environment, but in modern times are responsible for the high incidence of obesity and diabetes [114]. Many metabolic disorders have been shown to represent mismatches – in the way our bodies are regulated – between the austerity of the ancestral environment the cheap abundance of calories and fats seen in modern times.

The thrifty genotype hypothesis [115] posits that certain human genes that are associated with increased risk for metabolic disease today were naturally selected in the past because they helped their bearers to be more 'thrifty' with energy stores. According to this hypothesis, phenotypes that express low metabolic rates enjoy a survival advantage under deprived circumstances. However, they face increased risk of negative health consequences when sugars and fats are artificially abundant [116]. Thrifty genes, associated with a number of different metabolic traits, are thought to allow an organism to conserve calories, increase fat deposition and adopt a sedentary nature [117]. Interestingly, AD patients are well-known to exhibit many of these well-established thrifty traits, suggesting that genes that predispose to AD neuropathology may be part of a complement of thrifty genes that have a tendency to present comorbidly.

The metabolic syndrome, a suite of thrifty traits that tend to present together including obesity, diabetes and heart disease, has been strongly tied to AD [106]. Studies even show that AD patients have lower levels of physical activity and energy expenditure than age-matched elderly individuals that do not have AD [118,119]. Both old age and, especially, AD have been associated with lower testosterone levels, lower muscle mass, lower thyroid levels, lower growth hormone levels, insulin resistance, increased adiposity, upregulated hypothalamic pituitary adrenal axis and a significantly lower resting metabolic rate [67,68]. Because modern day individuals that develop AD show a highly increased propensity for developing these traits, we can expect that this was also the case in ancestral times, and that such individuals would have enjoyed increased survival under deprived circumstances.

The thrifty phenotype hypothesis has been used widely to interpret distinct patterns of the geographic distribution of genes that are associated with the metabolic syndrome. This widely accepted interpretation emphasizes that, populations of foraging individuals that live in areas where, until recently, food has been relatively unpredictable or frequently scarce have much higher prevalence of thrifty genes [120]. The traits that these genes code for helped these individuals survive during prolonged periods of scarcity by allowing them to maintain low basal metabolic rates [115]. Recent research has shown that the geographic distribution of the gene most strongly related to AD susceptibility – the APOE 4 allele – matches the geographic distribution for all the other major thrifty genes, such as insulin resistance and increased adiposity. This has influenced some researchers to hypothesize that APOE 4 may also be a thrifty gene [97]. These findings further indicate that AD may represent a type of thrifty phenotype that would have been well-suited for nutritional scarcity.

Type 2 diabetes mellitus (T2DM) has been characterized as a disorder that is a prototypic example of Darwinian medicine and the thrifty phenotype. It has been thought for decades now that, on a cellular and organ system level, the disorder represents a thrifty condition – insulin resistance – that would have only rarely manifested as disease in the ancestral environment because at that time individuals had no access to pure sugar or processed foods [115]. The current literature holds that insulin resistance, brought about by genes for T2DM, represents a finely tuned physiological state and that its cellular and molecular pathways have been refined by natural selection over millions of years to help the organism conserve blood sugar [117]. Not surprisingly, AD and T2DM share many common biochemical and physiological pathways. The uncanny resemblances include widespread changes in insulin signaling, changes in transforming growth factor systems, similarly high counts of hyperphosphorylated proteins, and dense aggregations of amyloid peptides [121]. Fascinatingly, there is a 90 percent structural similarity between the amyloid peptide in AD and the islet amyloid polypeptide in T2DM; this close correspondence in amino acid sequence is thought to suggest similar physiological roles ^122^. In addition, other peptides such as APOE, heparin sulfate proteoglycans and serum amyloid P play "crucial" roles in both T2DM and AD [123,124]. The literature on the adaptive functions of diabetes has never been reconciled with the literature on the similarities between AD and T2DM. A comprehensive comparison of pathology between the two should be informed by the hypothesis that AD is also a thrifty condition.

Each of the thrifty conditions discussed here have inherent drawbacks. The organism is calibrated to expend less energy, supply lines are narrowed and functionality is constrained. Every adaptation in evolutionary science represents a tradeoff where an interest in one concern is emphasized at the expense of another. The foremost, omnipresent concern for all life forms is "the struggle for sustenance" and the brain changes that accompany aging are clearly a response to this concern. Paradoxically though, these changes are being made at the expense of the human mind. The present hypothesis, though simple and straightforward, may remain hostile or inconceivable to many because of humanity's anthropocentric perspective on the mind. That the metabolic output of internal organs can decrease in response to starvation does not constitute a hostile idea. That the metabolic output in the "organ of thought" might also decrease, however, is unfriendly for many philosophical (Cartesian dualist) reasons. Perhaps the main reason that this is unfriendly is that many people have never considered that their thoughts arise from, and in every sense are made possible by, ingested food. We have come to revere our own minds so much that it is hard for us to see that advanced intelligence, like every biological adaptation, can be maladaptive under the right circumstances.

"Paradoxically, sometimes losing neurons can improve brain function, as happens when synaptic connections and neurons that have not been extensively used die off, in perhaps the most dramatic case of use it or lose it. Keeping unused neurons supplied with blood, oxygen and energy is wasteful, and getting rid of them keeps the brain more focused and efficient."

-Norman Doidge, 2007 [47]

It has been concluded that millions of years of drastic fluctuations in food availability would have exposed our close ancestors to periods of unavoidable hunger and starvation [64,112]. After sufficient time passes without a meal, the body's cells exhaust the supply of blood sugars and hypoglycemia ensues. When the nutrients from the last meal are depleted, the body begins to catabolize stored energy. Glycogen from the liver and muscles can sustain a moderately active human for several hours, but when these fuel stores run out the body must begin to burn supplies of fat. The process of ketosis liberates body fat for use in the muscles and brain; however, the brain has priority over these fuel reserves. Many tissues in the body can down-regulate their metabolic rate in the absence of energy, but the brain cannot [125]. Once the brain can no longer extract the fuel it requires from the blood, unconsciousness, coma and death ensue [126]. This shows that the disproportionately gluttonous human brain can potentially be an extravagant liability. Inability to temper this organ with age would have created a burden that our aging forebears could often not afford.

## Implications of the hypothesis

As longevity around the world continues to climb, the incidence of AD has been increasing steadily. Today, it is estimated that about four million people in the United States suffer from AD and that number is expected to triple before 2050 [127]. It has even been called the pandemic of the 21^st ^century [128]. In the environment of evolutionary adaptedness, our lives were far shorter and true cases of AD would have been extremely rare throughout prehistory. It is commonly pointed out that evolution has no foresight or predictive capacity and, unfortunately, it seems that an adaptation that was meant to help our ancestors now makes us susceptible today to senile dementia since we have artificially extended our lifespan.

It may not possible at this point to determine irrefutably if AD is a protective adaptation that served a purpose during human evolution. Further analysis and experimentation should allow more definite conclusions. For instance: (1) the formation of plaques and tangles should prove to be the most energy and resource efficient way for neurons to lose connections; (2) the neuropathology should present only in brain areas that are consistent with neuroecological rationale; (3) patterns of heritability and etiology should reflect evolutionary trends; (4) onset of pathology should coincide with ages of reduced foraging ability; and (5) molecular patterns and evolutionary signatures (such as relative intron-exon selection) should reveal that the genes involved in AD were positively selected relative to alternative alleles. The present hypotheses may also have major implications for medical research and ultimately for treatment: (1) the pathways that allow the reversal of hyperphosphorylated tau in starving rats will hold key clues to reversing or preventing AD (2) the close evolutionary association with diabetes mellitus and other thrifty disorders should influence researchers to focus on pathocomparative analyses (3) theorists now have a reason to reconcile the vast literature on thrifty genes, deprivation syndromes and phenotypic plasticity with the literature on the pathophysiology of AD.

The present evolutionary analysis of AD can be extended in far reaching ways. It is known that the most conspicuous of the various changes that accompanied human evolution was the significant increase in relative brain size, especially the size of the cerebral cortex. How human encephalization was accomplished and why hominids were selected for bigger brains despite the energetic costs has been a subject of much debate [129]. Many of the concepts invoked in this debate might help to elucidate the evolutionary basis for AD. Weighing the ecological costs of AD with the benefits might prove to be very informative, and future work should include discussion of concepts like carnivory vs. herbivory, dietary quality, encephalization index, the expensive tissue hypothesis, food extraction techniques, life history theory, optimal foraging theory, resting vs. maximal metabolic rate, total energy budget, the Kleiber relationship, evolutionary theories of aging and others.

Many species of marine animals called urochordates, also known as tunicates and sea squirts, do an amazing job of illustrating how intelligence and excessive nervous tissue can be an encumbrance that natural selection will go to lengths to protect against. This small invertebrate is born with sense organs and a brain (a cerebral ganglion) and resembles a tadpole. It needs its simple nervous system in order to coordinate its behavior while searching for a home early in life. The sea squirt has a second stage where it locates a suitable surface on a rock and attaches itself permanently. From then on, its life history changes from a mobile to a sedentary strategy, and it actually spends the rest of its life passively filter feeding – grabbing nutrients as they float by [130]. After attaching to the rock, the little creatures digest their brains and large regions of their own nervous systems specifically in order to minimize energy use [131]. They never grow them back.

Similarly, individuals in ancestral hunter-gatherer tribes in their 30s, 40s and 50s must have experienced diminishing usefulness for higher order intelligence. They were no longer fastidiously busy creating novel motor praxes, learning foreign spatial maps or developing original motor activities. The pressure on them to internalize unfamiliar information and to see unprecedented relationships between important variables dwindled every year of their life as their experience increased. They were not actively developing untried social skills to create new alliances or find new partners for copulation, and also were not directly responsible for caring for infants or inculcating children. Further, they could no longer employ many of the high yield foraging techniques that they utilized in their youth, so they must have been quite receptive to the benefits of adopting an alternate "thrifty cognitive" strategy.

A thoughtful Talmudic commentary states that "scholars become wiser as they age, but the noneducated become foolish." Specialists in technical fields acquire large amounts of knowledge over the years that, during old age, allow them to converse fluently and intelligently on their subject despite the cognitive morbidity that they may suffer. In a general sense, these sentiments reflect the idea that whatever you spend much time doing, you become an expert at. Much unlike today, every individual in prehistoric groups foraged throughout the day from a very early age until death. The expertise gained from this would have made them very "wise" at hunting and gathering. It is the view espoused here that this physical and mental wisdom, earned from decades of foraging, allowed foragers to reduce their investment in the 'fixed cost' of energy consumption within the brain. Moreover, it is clear that preclinical AD would not have been disruptive to the implicit techniques, procedural knowledge or motor praxes necessary for foraging, even though clinical AD is disruptive to the explicit techniques, semantic knowledge and declarative cognition necessary for both occupational employment and autonomous living today.

## Competing interests

The author declares that they have no competing interests.
